# Analyzing Teacher–Student Verbal Interaction in Elementary Chinese Comprehensive Class: Insights from Flanders Interaction Analysis System

**DOI:** 10.3390/bs15040429

**Published:** 2025-03-27

**Authors:** Xingrong Guo, Wensi Yang, Yiming Guo

**Affiliations:** 1 College of Foreign Languages, Shanghai Maritime University, Shanghai 201306, China; 202130810010@stu.shmtu.edu.cn; 2 Shanghai Normal University Affiliated Jinshan Longhang Primary School, Shanghai 201508, China; 3 School of Economics and Management, Shanghai Maritime University, Shanghai 201306, China

**Keywords:** Flanders Interaction Analysis System, Chinese as a foreign language, interaction analysis, classroom discourse

## Abstract

This study analyzes the behavioral characteristics of interactions between an experienced Chinese teacher and students in an elementary Chinese classroom through a case analysis, aiming to provide insights for novice teachers. Using the Flanders Interaction Analysis System (FIAS), this research evaluated interaction patterns in a 45 min Chinese demonstration class involving eight students from European, American, and Southeast Asian countries. The results showed the following: (1) The teacher’s speech accounted for 44.72% of class time, and they mainly used direct language to guide students. (2) Students’ speech accounted for 41.27%, showing their active participation but limited autonomy in self-initiated talk and independent engagement. Improvements are needed in students’ ability to initiate more speech and engage independently without teacher prompts. (3) Classroom structure reflects “student-centered, teacher-led” principles through open questioning and peer interaction. This study proposes some practical recommendations, advocating for increasing opportunities for student-initiated discourse, implementing structured questioning techniques, and strengthening collaborative group work. These findings provide empirical insights into balancing teacher–student interaction dynamics in cross-cultural language classrooms.

## 1. Introduction

With the globalization of Chinese language education, more and more scholars are paying attention to empirical research on real classroom teaching. Student-centered learning (SCL) has become a focus in Chinese teaching research. Weimer and Sharkey (2003) defined this approach as emphasizing learners’ active role in knowledge construction, which involves collaborative dialog and independent decision-making. SCL aligns with communicative language teaching principles that emphasize learner agency in target language use (Nunan, 2012; Richards, 2005). The synergy of these theories and methods makes SCL an important framework for promoting autonomous learning in cross-cultural Chinese classrooms.

Research on teaching methods for Mandarin began in the late 1960s. For example, Wrenn (1968) explored teaching methods in the American educational environment. As the main training base for Chinese educators worldwide, this classroom environment research provides important observational data for analyzing cross-cultural teaching practices. McGinnis and Ke (1992) proposed a pedagogical framework that incorporates culturally authentic resources into the teaching of Chinese literacy as a second language, establishing foundational practices for text-based cultural edification. The academic progress of Chinese teaching research has gradually enriched the analytical framework. Lv (2004) suggested the use of multiple modes to systematically analyze the teaching process in the Chinese classroom. Han (2018) studied the implementation of task-based teaching strategies in teaching Chinese as a foreign language. Cheng (2013) conducted a microanalytic study exploring the interaction between classroom interaction patterns and bilingual communication practices, employing conversational analysis techniques to decode linguistic alternation phenomena in second-language acquisition contexts. L. Zhang (2018) conducted a comprehensive analysis of spoken language pedagogy within Chinese as a Foreign Language (CFL) instruction, formulating theoretical frameworks to develop instructional competencies among undergraduate students specializing in international Chinese education. Jiang (2020) investigated the implementation of inverted classroom methodologies to optimize pedagogical outcomes in cross-cultural Mandarin teaching contexts. Contemporary research methodologies demonstrate increasing sophistication in addressing linguistic challenges. W. Wang (2022) employed an experimental phonetics methodology to develop targeted interventions for Mandarin tonal acquisition in second-language instruction. Parallel innovations have emerged in pedagogical design, as evidenced by Kim et al.’s (2024) examination of schema-based instructional frameworks, which confirmed their efficacy as a pedagogically robust approach within the context of Mandarin acquisition.

Contemporary pedagogical practice necessitates educators’ strategic implementation of multifaceted instructional approaches to optimize knowledge transfer processes. Didactic dialog constitutes a pivotal element within these pedagogical processes, as substantiated by Reeve et al. (2004). Scholarly investigations within Mandarin pedagogy have systematically investigated multiple dimensions, spanning digital learning environments to interpretive challenges in communicative exchanges. These academic inquiries have collectively advanced the comprehension of instructional dynamics in Chinese language acquisition contexts.

According to Reeve et al. (2004), verbal interaction between teachers and students is very important to pedagogical dynamics. The Flanders Interaction Analysis System (FIAS), developed in 1968, quantifies verbal behaviors using coding protocols and matrix analysis and has become a foundational tool in classroom interaction research. J. Liu (2016) and Huang and Zhu (2019) identified this approach as the main tool for examining pedagogical interactions across global education, constituting a standardized observational framework for cross-cultural instructional studies. The methodology demonstrates cross-cultural applicability through global implementation. Illustrative studies include Smith et al. (1977)’s systematic investigation of physics educators’ communicative strategies in American higher education institutions, complemented by Roberts (2009) analysis of ethnolinguistic variations in instructional delivery patterns across demographic groups. Binmohsen and Abrahams (2020) conducted a comparative analysis of digital and traditional science teaching methods, making use of this analytical framework. Riyadini and Basikin (2024) made innovative contributions by establishing correlations between metrics of communicative engagement and the development of English language proficiency in second-language instruction. The application of the FIAS in the Chinese academic field began relatively late. Fundamental research in this area started in the late 1980s and mainly focused on theoretical adaptation and classroom implementation trials. H. Zhang (2014) conducted fundamental research that comprehensively probed into the operational variables and diagnostic characteristics of the FIAS. G. P. Yang and Zhang (2018) used this framework to methodically assess ten exemplary teaching cases, pinpointing the distinctive traits of pedagogically successful lessons. Liang (2020) furthered this methodological advancement. Their comparative analysis of interrogative patterns between seasoned and novice secondary mathematics teachers used the FIAS to measure discourse differences.

Although widely applied in language education, the FIAS has not been well studied in CFL classrooms. Existing FIAS studies in CFL contexts mainly focus on East Asian learners (e.g., Liang, 2020), paying less attention to multicultural classes with students from different language backgrounds. Previous findings about “teacher-centered” patterns (Huang & Zhu, 2019) also lack an analysis of how specific teaching methods affect student participation. The methodologies used to analyze teacher–student interactions in this specific context have not been fully studied. This study aims to address this gap by analyzing teacher–student interactions in a multicultural CFL classroom, thus contributing to the understanding of how verbal interaction patterns impact learner autonomy and participation. This study seeks to address three research questions:

(1) How does teachers’ behavior influence the cultural–linguistic backgrounds of elementary CFL learners’ autonomous participation?
(2) What verbal interaction patterns occur in multicultural CFL classrooms?
(3) What are the illuminations for enhancing the classroom teaching of novice Chinese language teachers?

## 2. Research Methodology

This study used the qualitative case study method and FIAS to analyze the teacher–student verbal interaction in a CFL classroom. Teacher A in this study was a woman with 17 years of experience teaching CFL, particularly in multicultural classrooms. The FIAS was used to analyze a 45 min lesson and created 900 coded intervals (3 s intervals) to record interaction details. Although analyzing one lesson may limit generalizability, it aligns with the goal of finding typical interaction patterns. The methodology is justified according to Gerring (2007). Quantitative approaches, especially FIAS-based interaction measurements, offer practical strategies for new teachers by converting abstract teaching concepts like student-centered learning into quantifiable benchmarks.

### 2.1. Participants

The participants in this study were from a teaching video of an exemplary comprehensive lesson on elementary Chinese as a foreign language. The video was used to teach Mandarin to eight foreign students from Europe, America, and Southeast Asia. These students were at the elementary proficiency level and had a vocabulary of around 200 words. The main teaching material used in the course was Lesson 15 of New Concept Chinese, published by Beijing Language and Culture University Press in 2012, titled “Does This Red Dress Look Nice?”.

This video is available on multiple platforms, such as the website for publishing reference cases of International Chinese Language Teaching classes, and Bilibili, a leading video platform in China. It is designated as a demonstration course for teaching Chinese as a foreign language. According to Bilibili’s statistics, from October 2020 to March 2024, the video accumulated 129,000 views. Its educational value is reflected in the following evidence: (1) an extremely high engagement rate (5298 favorites, 4.1% save-to-view ratio, 421 bullet comments); (2) widespread application in teaching practice, with positive feedback from 158 CFL teachers, including remarks such as “This video serves as a model training course for CFL instruction at Chinese universities”, and high praise for its refined instructional design. This video has a large amount of viewing data, indicating its strong demonstration effect and high teaching inspiration value. The reasons for choosing this course are as follows:

(1) This class is an exemplary lesson from New Concept Chinese, a standard textbook in global Chinese programs. Moreover, it has been annotated as a lesson from the “International Chinese Language Teaching Reference Cases—Elementary Comprehensive Course” published by Beijing Language and Culture University Press. This course effectively demonstrates the core objectives of elementary-level comprehensive curricula, including supporting vocabulary acquisition, sentence structure exercises, and cultural understanding development.

(2) The learner profiles in this video are multicultural. The class consists of eight international students with an elementary level of Chinese proficiency from Europe, America, and Southeast Asia. This multicultural environment reflects the typical cross-cultural communication dynamics in CFL classrooms, characterized by diverse linguistic backgrounds (Zou & Liu, 2015), adaptation to participatory norms (Claramita et al., 2022), and the use of scaffolded interaction strategies (Reimer, 2022). It enables an exploration of how teachers balance instructional control and learner autonomy in culturally diverse settings.

(3) The course is marked as a student-centered teaching model, reflecting the best practices of CFL education. The video of this lesson is sourced from the International Chinese Language Teaching Reference Cases. The teacher uses multimodal teaching strategies, such as visual aids, role-playing, and situational practice exercises. These strategies reflect the “student-centered, teacher-led” method, providing real observable data on interaction types.

(4) This widely viewed demonstration lesson addresses common challenges faced by novice teachers, such as low interaction efficiency and insufficient student participation (T. M. Wang & Ren, 2015). The case analysis provides practical techniques for improving verbal interaction patterns in CFL classrooms.

Ethical review and approval were waived for this study in accordance with local legislation and institutional requirements, as the object of this study was a publicly released teaching video of an exemplary comprehensive lesson on elementary Chinese as a foreign language conducted by a female teacher, which was available for free viewing. All identifiable student information was anonymized in the transcriptions.

### 2.2. Data Analysis

The data were documented in a classroom observation sheet. Each row of the sheet represented a three-second observation interval, and the columns corresponded to different time points within the 45 min lesson. To ensure consistency, two researchers independently coded the video.

After coding, this study conducted a sequence matrix analysis, including paired continuous coding, to identify recurring teacher–student interaction sequences. It could also determine who led classroom discussions by calculating the total number of conversations between the teacher and students to compare the frequency of their conversations.

Finally, a second researcher repeated the coding process, and a reliability check was performed to guarantee data consistency. Through iterative calibration, the two trained coders achieved an inter-rater reliability of Cohen’s κ = 0.82. Discrepancies (less than 5% of the codes) were resolved through consensus discussions.

### 2.3. Research Design

Ning and Wu (2003) and H. Zhang (2014) provided a summary of the Flanders Interaction Analysis System, which consists of a set of codes that characterize classroom verbal interaction behaviors, a set of predefined criteria for observing and recording the codes, and a matrix table for displaying data, analysis, and achieving research goals. These components allow for organized research into classroom communication patterns, helping researchers study how teachers and students interact. Table 1 shows how the FIAS classifies classroom conversations with specific codes and descriptions.

Table 1 lists the coding scheme, which categorizes verbal interaction behaviors into three broad types: teacher language, student language, and silence or confusion (encompassing no effective verbal activity). These 10 interaction types are numerically coded from 1 to 10 (Freiberg, 1981). The system assigns seven codes to teacher-initiated exchanges, two to student participation, and one to non-verbal periods.

### 2.4. Classroom Observation and Data Recording

The first step in analyzing the teaching video was to watch the entire video and understand the teaching process. Then, we used a timer to pause every three seconds and record the teacher–student behavior (Septiningtyas, 2016). Each observed operation received a code that matched the classification system. A code corresponding to the coding scheme presented in the previous section was assigned to each observed behavior, and these codes were recorded for subsequent analysis. All the codes were then entered into a classroom observation record sheet in chronological order based on the time of observation. For future analysis, these codes were recorded horizontally. Finally, there were a total of 900 codes, as shown in Table 2.

As shown in Table 2, all codes were entered into a classroom observation sheet. The horizontal rows represent the teacher’s or students’ behaviors every 3 s.

Each row records 20 sequential behaviors (with each entry corresponding to 3 s). The vertical columns correspond to the 45 min class duration. After coding all 45 min of the video content, another researcher was invited to verify the coding for accuracy. After discussion among the researchers regarding the uncertainties, the final data presented in Table 2 were determined.

### 2.5. Construction of the Analysis Matrix

The corresponding data matrix was constructed based on the coding of the classroom observation record form. The specific matrix model is presented in Table 3.

As shown in Table 3, a matrix model was derived from coding classroom observation records. It was created by pairing consecutive codes to form sequential pairs and then counting the frequency of identical pairs. The total numbers along Table 3’s edges count how many times each behavior appeared during the 45 min class, providing an overview of the structural characteristics of classroom communication dynamics.

**Figure 1:**
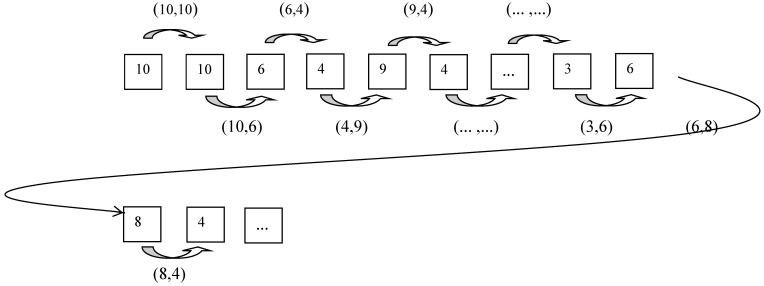
Schematic diagram of coding sequence pairs.

Figure 1 shows a schematic diagram of the coding sequence pairs.

As shown in Figure 1, the first step involves creating sequential pairs by combining every two adjacent codes. For example, if the first row of Table 2 contains 20 codes, then there will be 19 sequential pairs in the first row, followed by the last code in the first row and the first code in the second row, as a set of sequences. This process continues for the entire table.

Subsequently, the number of identical pairs is determined and filled in in the corresponding cells of the matrix. For example, if there are 13 sequential pairs of (8, 4), then the number 13 is in the table in the 8th row and 4th column. Similarly, if there are 17 sequential pairs of (4, 8), then the number 17 is filled in in the corresponding place of the 4th row and 8th column. Higher values in the matrix indicate more frequent behavioral sequences. The values of each row and column are tallied independently, and the result represents the total number of codes recorded within the 45 min course.

## 3. Results

**Figure 2:**
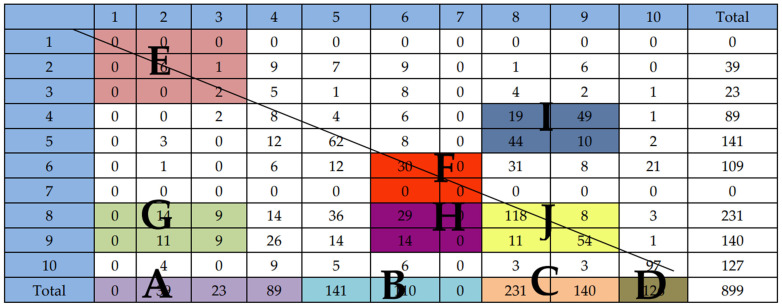
Matrix of teacher’s and students’ speech acts in foreign Chinese classroom.

According to Flanders (1963), specific behaviors represented by the cells within the matrix possess distinct characteristics. Figure 2 shows the matrix of the teacher’s and students’ speech acts in the CFL classroom.

As shown in Figure 2, the numbers on the diagonal represent continuous actions with a tracking duration of more than three seconds. The bottom four sections (labeled A, B, C, D) show the total number of connected code pairs in each category. These values helped track how often the teacher and students communicated and their interaction patterns.

### 3.1. Matrix Analysis

Area E contains nine sequential pairs of cells, which is termed the positive integration grid, indicating good teacher–student interaction behaviors. In region E of Figure 2’s matrix, there are nine instances of interactive behavior. During class, teachers often offer praise or encouragement in short phrases, which may not be recorded if they last less than 3 s. As a result, sustained praise behaviors were not frequently documented, leading to a low count in region E. However, this does not necessarily suggest a lack of positive teacher–student verbal interactions in the classroom. Throughout the video, the teacher emphasizes students’ reactions to praise and occasionally accepts their ideas by paraphrasing.

Area F consists of four cells indicating direct teacher instruction, such as issuing commands or directives and criticizing or expressing authority. In the F zone, only 6–6 appeared 30 times, and there were no sequential pairs related to 7. This indicates that the teacher refrained from condemning students’ behavior in class, students did not challenge the teacher’s authority, teacher–student interaction was cordial, and the classroom atmosphere was active. However, it is evident that students’ autonomy was limited, and they relied on the teacher’s guidance for appropriate actions.

Areas G and H show that the teacher either directly or indirectly ended students’ speech in the classroom. Video observations revealed that Teacher A preferred to interrupt students in the classroom shortly after they completed speaking or at the end of their speech, allowing students maximum opportunities to speak before proceeding with the lesson content.

Area I indicates teacher–student question-and-answer sessions. In Figure 2, the frequencies of (5, 8) and (4, 9) exceed 40. Specifically, (5, 8) occurs 44 times, reflecting the teacher’s practice of having students read aloud after teaching. The frequency of (4, 9) is 49, surpassing that of (4, 8). These results emphasize the importance of cultivating students’ independent learning abilities and encouraging them to speak up to increase their learning interests, which Teacher A emphasized during the course.

Area J shows that students engaged in extended statements or continuous communication, with (8, 8) occurring 118 times during the video observation. This can be attributed to teacher guidance, frequent practice, and students’ consistent follow-up, rather than students having inherently long responses. Additionally, (9, 9) occurred 54 times, suggesting that students took the initiative to raise their hands, come to the front of the podium, and make presentations, rather than just giving brief answers. Through this process, students developed their verbal expression and paragraph-constructing abilities.

### 3.2. Ratio Analysis

#### 3.2.1. Analysis of Classroom Structure

Flanders (1961) identified three primary types of verbal interaction behaviors in the classroom. By analyzing these categories, the proportion of each category in the classroom can be determined. This reflects the classroom’s structure. The sum of the numbers in columns 1 through 7 in the matrix represents the entire number of teachers’ verbal behaviors. The ratio of teachers’ verbal behaviors to the overall classroom behaviors indicates the proportion of teachers’ speech in the class.

Multiplying the sum of columns 1–7 by 3 gives the total duration of teacher language in the classroom (in minutes). Similarly, the number of student verbal interactions, the ratio of student language, and the duration of student language can be calculated, enabling an analysis of the lesson’s structural features. Table 4 shows the results of the classroom structure analysis. By counting the occurrences, proportions, and durations of teacher language, student language, and invalid language, the structural characteristics of the classroom are revealed.

Table 4 shows that in the recorded elementary Chinese conversations, the teacher spoke 402 times (20.1 min, 44.72% of the class time), while the students spoke 371 times (18.6 min, 41.27%).

Compared to the typical classroom language distribution (Gao, 2009), where teacher discourse typically accounts for 68% and student discourse for 20%, the student participation rate for this lesson was double the average. These indicators show that the curriculum achieves an enhanced interactive balance through intentional instructional design that goes beyond traditional participation models.

Ineffective language appears 126 times in the video (6.3 min accounting for 14.01% of the total duration), including group discussions among classified students. However, through efficient time management and structured content delivery, the course maintains structural productivity. The observed teacher–student verbal communication ratio is 1.08, which is consistent with the optimal classroom dynamic ratio of 1–2 recommended by C. Y. Yang and Yan (2010). This indicator indicates that learners’ participation and communicative initiative was improved in the course.

In summary, this demonstration lesson was well–structured, with students actively participating in large numbers. The classroom environment promoted active, co-constructed learning between the teacher and students. The classroom structure was teacher-led, yet students also had a high level of initiative, and the classroom activities were co-constructed by both the teacher and students.

#### 3.2.2. Analysis of Teaching Tendencies

##### Control Type Analysis

Columns 1–4 and columns 5–7 in the matrix represent the direct and indirect effects of the instructor’s words, respectively. By summing the two sets of values for indirect and direct influence and their ratios, the teacher’s tendency to exert control over the classroom becomes evident. Table 5 shows the analysis results of the types of teacher control.

Table 5 shows that the teacher’s indirect language, including praise and questions, accounted for 37.57% of the teacher’s verbal communication. In contrast, direct language, such as lecturing and instructions, accounted for 62.43%. The ratio between these two types of language was 60.18%, which is less than 1.

Although Table 5 suggests that the teacher tended to rely on direct language to influence students in classroom teaching, the video evidence of teaching practices suggests that this is different from traditional methods. Teacher A adopted both the teacher–student and student–student interaction, using objects or pictures to visually help students understand vocabulary and grammar. In addition, rich practice scenarios were provided to enhance students’ mastery of the material. This blended methodology promoted active learning participation while maintaining teaching efficiency to maximize target-language production per instructional minute (Long, 2015). This is critical in CFL contexts where limited contact hours demand optimal time allocation between linguistic input and interaction.

##### Analysis of Reinforcement Types

Regarding reinforcement, columns 1–3 of the matrix demonstrate the teacher’s positive reinforcement’s influence on students, while columns 6–7 represent negative reinforcement. As posited by Ning and Wu (2003), when the ratio of positive to negative reinforcement is greater than 1, positive reinforcement is dominant; when it is less than 1, negative reinforcement prevails.

The video shows that the teacher offered praise and encouragement to students through expressions such as “good”, “very good”, “right”, and “you said very good”. However, due to their short duration (less than 3 s) and difficulty in accurate counting, only 39 instances of praise were recorded. Table 6 shows the types of teacher reinforcement. By counting the number of occurrences and the ratio of positive reinforcement and negative reinforcement, it evaluates the impact of the teacher’s reinforcement behaviors on students.

Table 6 shows that Teacher A produced 62 positive feedback instances versus 110 corrective guidance instances. The ratio of positive to corrective reinforcement was 56.36%. Classroom observations demonstrated frequent motivational techniques and recognition of student contributions, establishing supportive learning conditions. Teacher A systematically validated students’ perspectives by restating or expanding their responses in class participation. Corrective interventions mainly involved providing activity guidance and targeted support for learners who encountered communication difficulties, avoiding evaluative criticism. Since students often need teacher guidance before actively answering questions, in some cases, they may respond with limited autonomy. Therefore, the above analysis indicates that negative reinforcement dominated the teaching style in this lesson. Through proactive guidance, students became increasingly engaged, raising their hands to speak in teacher–student or peer interactions, demonstrating greater confidence. This helped to create a positive classroom atmosphere, in which empirical studies have shown a strong link between a positive environment and increased student engagement. Favorable conditions, such as supportive teachers and safe learning spaces, enhance academic performance and personal development (Qi, 2024).

#### 3.2.3. Feature Sequence Pair Analysis

In the matrix table, some sequential pairs have certain similarities, as shown in Table 7 and Table 8. According to J. S. Liu (2019), the frequency and ratio of these sequential pairs can reflect the characteristics of teachers’ teaching behaviors.

As shown in Table 7, the sequential pairs (4, 4), (4, 8), (8, 4), and (8, 8) form a closed-type pattern, wherein the teacher poses questions and students respond passively. The code pair (4, 4) indicates the teacher’s question; (4, 8) represents the teacher’s question followed by a student’s passive response; (8, 4) signifies a student’s passive response followed by the teacher’s subsequent question; and (8, 8) denotes a student’s passive response.

Table 8 shows the open-ended question and answer table for teachers and students. The code pair (3, 3) indicates accepting or using a student’s claim; (3, 9) represents the teacher accepting or using a student’s claim followed by a student’s initiative response; (9, 3) signifies a student’s initiative response followed by the teacher’s acceptance or use of the student’s claim; (4, 9) denotes the teacher’s question followed by a student’s initiative response; (9, 4) means a student’s initiative response followed by the teacher’s question; and (9, 9) shows a student’s initiative response.

The questioning format in this classroom departed from conventional teacher-led exchanges, where students react passively to closed-ended prompts. Aligned with Reznitskaya and Maughn’s (2013) dialogic teaching framework, this open response through inquiry prompts has been shown to significantly enhance cognitive engagement in L2 classrooms (Nunan, 2012). Instead of adopting an open-response framework that allows multiple valid responses, this approach encourages learners to formulate individualized replies through active engagement with inquiry prompts, requiring cognitive participation rather than mere recitation. The sequential pair (9, 9) appears 54 times, indicating that many students consistently answered open-ended questions actively, either by raising their hands or answering immediately after the teacher posed the questions. This demonstrates that students were actively involved in learning and acquiring knowledge and skills in response to the teacher’s open-ended questions.

Upon comparing Table 7 and Table 8, it is noted that the (4, 8) sequential pair appears 19 times, the (8, 8) sequential pair appears 118 times, the (4, 9) sequential pair appears 49 times, and the (9, 9) sequential pair appears 54 times. This indicates that in the elementary CFL class shown in the video, the teacher asks questions frequently. The question-and-answer pattern deviates from the traditional model. The (9, 9) sequential pairs, which occurs 54 times, involves non-predetermined, open-ended questions that allows students to freely express themselves, either by raising their hands spontaneously or answering together immediately after the teacher asks. This further validates students’ active engagement in learning and knowledge acquisition in response to the teacher’s open-ended questions.

In addition, the video shows a small-class teaching format with only eight foreign students. Some questions are addressed to the whole class, while others are directed at specific students, ensuring each student has the opportunity to be called on to answer. This significantly increases student participation and learning motivation. Teacher A allows students to assume the teacher’s role at the podium, in addition to the traditional model where the teacher asks questions at the podium and students respond from their seats. For example, when practicing the colors of national flags, the teacher purposefully assigns each student their respective national flag, enabling them to answer questions and improve their confidence, better reflecting the “student-centered” principle.

## 4. Discussion

This study examines classroom interaction patterns through a detailed analysis of a demonstration class. Although focusing only on one case limits broader conclusions, this method generated a large amount of data (900 code exchanges). Studying high-quality teaching examples remains a methodologically rigorous approach for identifying effective practices, as advocated by Flyvbjerg (2006) who demonstrated case studies’ unique capacity to generate context-rich pedagogical knowledge. These results are consistent with early FIAS research on student-centered teaching. However, when comparing grammar-based courses with culture-focused courses, the results may differ. Subsequent research can test this method in different types of classes to improve its wider application.

### 4.1. Influence of Teacher’s Verbal Behavior

According to the FIAS statistics, columns 5–7 in the matrix table (Figure 2) reflect the direct influence of the teacher’s speech. Since the teacher’s lecture and directive discourse in the classroom were more frequent, such as showing a video of the text, making students read aloud, and guiding them to answer questions, they all belonged to the form of direct language. Direct language accounted for 62.43% of the teacher’s speech acts, and the teacher spent most of their time lecturing, with a percentage of up to 35.07%, meaning that in the lesson, the lecture time was relatively long. It was acceptable given that the teacher had to provide the best possible content explanations for the students to fully comprehend the teaching material.

Therefore, the method used by the teacher was mainly “lecture”, resulting in limited student autonomy in the classroom and a passive state of receiving knowledge (Toyama & Yamazaki, 2021). The statistics showed that the component of indirect influence, including asking questions, came in first, with a proportion of over 20%. However, the information obtained from the video recording shows that the teacher purposefully asked questions and anticipated receiving an answer from the class. However, in this teacher-led classroom, there was less ineffective language and confusion in the teaching process and the overall pace of the classroom was more efficient.

Teacher speech occupied 44.72% of the classroom time, suggesting a teacher-centered dynamic that might have impeded students’ ability to initiate discussions. Such a high proportion of instructor-led communication could limit learner autonomy by reducing opportunities for independent idea expression and autonomous learning.

During a group activity where students described objects using new vocabulary, notable differences in participation emerged. Some students took leadership in peer discussions, while others were hesitant and sought the teacher’s help. The latter group struggled with target language output and spontaneous verbal contributions, indicating a reliance on teacher cues when lacking language confidence.

Educator responses include both positive and negative types, which influence learners’ behavior and independence. During instruction, teachers frequently employ encouraging comments like “Well done!” or “Good job!” to validate accurate responses. This encouragement not only motivates students but also provides emotional reinforcement for language acquisition. While error correction enhances precision, excessive intervention without self-correction opportunities may diminish learner autonomy. Striking a balance between supportive encouragement and targeted error correction emerge as critical for cultivating independent learning skills.

### 4.2. Features of Students’ Verbal Behavior

Columns 8-9 of the matrix Table 3 show how frequently students responded to questions in the class. Among these responses, the frequency of passive speaking was 231, while active speaking occurred 140 times. Overall, students’ speech acts accounted for 41.27% of the classroom time, with students being more active in answering questions in the class. Both initiation and response received greater percentages, indicating that the teacher gave ample opportunity for pupils to express their ideas using their own words. Responses and initiators were primarily generated by students. However, the frequency of response was 91 times less than that of initiation. This may be due to the fact that the target students in the teaching video were international students at the elementary Chinese level, and their ability to express themselves was somewhat restricted (Cai et al., 2021).

The analysis revealed that students demonstrated active participation, but their self-directed communication remained limited. Observation data indicated that learners initiated more conversational exchanges than they sustained. This suggests that students’ willingness to engage may have been hindered by linguistic proficiency gaps or cultural communication norms. This pattern highlights the importance of implementing pedagogical strategies that specifically promote spontaneous verbal initiation while establishing nurturing interaction frameworks for progressive dialog development.

Classroom observations show that Teacher A often asked closed-ended questions, such as “What color is this piece of clothing?” or “Do you like this color?” These types of questions usually received short and factual answers, thereby limiting the complexity of the conversation. On the contrary, open-ended prompts such as “Why do you think this color looks good?” encouraged students to make greater contributions. This comparison emphasizes the crucial role of strategic problem design in cultivating communicative autonomy. Closed-ended questions help to quickly check knowledge, while open-ended questions promote deeper understanding by inviting individuals to explain and elaborate.

The participants in this video were at the beginner level of Chinese proficiency and their ability to express themselves in the target language was significantly constrained by limited vocabulary, complex grammatical structures, and a lack of self-confidence. These challenges likely contributed to their passive engagement patterns, which manifested as responsive engagement with educator prompts rather than spontaneous dialog generation or autonomous opinion formulation.

In addition to language proficiency, learners’ cultural foundation further shapes their ability for self-directed learning. Many students come from an education system led by teachers that emphasizes passive knowledge absorption. As observed by Claramita et al. (2022), this cultural adaptation creates barriers to cooperative learning structures that require active participation and self-directed communication for adaptation.

In this case, participants come from different countries and explore the influence of cultural backgrounds on teacher–student interaction. Teachers need to pay attention to the influence of cultural differences on students’ performance in the classroom, design open-ended teaching situations, and arrange group learning for students from different countries. When they encounter difficulties in communication, teachers should help students organize their language and create an inclusive and diverse teaching atmosphere.

As a result, when a teacher asks specific questions or provides instructions, students respond more actively, but their sense of autonomy still needs to be strengthened. Additionally, this study found that the teacher paid much attention to the students reading aloud in the classroom, and when the teacher led the students to read aloud, they often requested for the students to repeat several times and practice continuously. This indicates that the teacher also paid close attention to fostering the oral expression ability of the international students at the elementary level.

### 4.3. Principles of Classroom Teaching

In contrast to the teacher’s unilateral output, the classroom in the instructional video has a comfortable and lively atmosphere where students can ask questions and engage in discussion with the teacher or the entire class at any time. At the same time, the classroom format is innovative, based on the traditional one (Hu et al., 2016). The teacher chooses pictures or videos to show the class content vividly, which can attract students’ attention and help them better understand the material. The interaction between teachers and students is not a mechanical question-and-answer format but a set of open-ended questions designed to encourage students to express their own views actively, reflecting the “student-centered, teacher-led” teaching principle.

Additionally, the teacher pays attention to student–student interaction, allowing students to approach the podium to play the role of the teacher, respond to inquiries from their peers, and ask questions to other students. Even the teacher plays the role of a student and answers questions, so that students are more willing to actively participate in the classroom and collaborate with the teacher to create learning activities. This increases students’ enthusiasm for learning and their willingness to actively participate, which is significant.

## 5. Conclusions

This study analyzes teacher–student language interactions in a Mandarin class teaching environment through the case study method. It highlights the value of a “student-centered, teacher-led” approach. The findings stress the importance of strategic questioning and feedback to boost student engagement. Practical recommendations for novice teachers include fostering autonomy and creating inclusive classrooms.

### 5.1. Practical Recommendations for Novice Teachers

Novice teachers should focus on teacher–student and student–student interaction styles to reduce the unnecessary repetition of knowledge points. More time should be allotted for students to practice language exercises. Teachers can design group discussions to enrich classroom activities, stimulate students’ communication with others, and enhance their autonomy. When planning such exercises, teachers should consider the students’ language proficiency level to organize the language activities appropriately.

In the classroom of this study, the teacher adhered to the “student-centered, teacher-led” teaching principle. The FIAS analysis shows that the distribution of the teacher’s speech acts and students’ speech behaviors was relatively balanced, complying with the ideal classroom structure of a balanced teacher–student verbal ratio. The data also show that students were actively engaged, accounting for 41.27% of the total speech, creating a lively classroom environment throughout the lesson. The fact that the students were engaged and eager to participate in every aspect of the lesson, and took on their primary role, not only met their needs but also increased the effectiveness of the teacher. It made teaching Chinese as a foreign language more vivid and engaging and inspired students to fall in love with learning the language and China.

Moreover, in video-based teaching, many teachers commonly use simplistic praise terms like “good”, “very good”, “right”, or “you said very well”. However, these lack a detailed assessment of students’ responses and are insufficient for promoting student improvement. To offer more effective feedback, teachers should analyze students’ responses and use more specific language for encouragement. For example, remarks such as “your pronunciation is very standard”, “you read the text very fluently”, or “your intonation is very accurate” can highlight aspects of students’ performance, in line with the findings of Reznitskaya and Maughn (2013). When students respond incorrectly, teachers should avoid using critical language. Instead, they can encourage self-correction through rhetorical questions or by repeating students’ statements. Novice Chinese teachers are encouraged to study and apply this “student-centered” approach in real-life Chinese classroom settings.

### 5.2. Implications for Curriculum Development and Teacher Training

During curriculum development and teacher training, it is encouraged to pay attention to the interaction between teachers and students and the way that students interact. Effectively designing teaching and advocating practical training that highlights student-centered teaching is very important. New teachers are encouraged to stimulate participation and critical thinking activities to cultivate students’ autonomy. Reimer (2022)’s research on effective teaching strategies also emphasizes the importance of well-balanced teacher–student interaction.

It is very important to allocate more time for students to practice language in terms of classroom interaction and time arrangement. Teachers can design group discussion, improve the atmosphere of classroom activities, encourage students to communicate with peers, and improve students’ autonomy. When designing these exercises, teachers should consider students’ language level to properly organize language activities.

In addition, teachers should increase the use of open-ended questions. This mainly encourages critical thinking, enables students to explore topics independently, and strives to build a student-centered and intellectually stimulating learning environment.

### 5.3. Limitations and Future Research Directions

Although this study provides some valuable suggestions for teacher–student language interaction in CFL classrooms, there are also some limitations.

(1) The small sample of eight students in a single instructional session restricts the generalizability of the findings. Future research could include more participants and diverse lesson formats to improve the broad applicability of the results.

(2) This study mainly focuses on a video recording, which also has certain limitations. Although video recordings can provide in-depth analysis of verbal communication, they may not fully capture the emotional and nonverbal signals that are important for assessing learner autonomy. For example, microexpressions, body posture, and other nonverbal cues can all affect the level of student engagement in interaction. Future research should shift its focus toward multimodal teacher–student interaction classes to address this gap.

(3) The class in this study is a demonstration lesson for teachers, aimed at showcasing the best designed classroom, which may deviate from the conventional teaching mode and reduce the applicability of observing interactive dynamics in the real world. Future research should concentrate on observing authentic classroom settings to rectify this limitation.

(4) This study does not explore the longitudinal effects of interactive patterns on students’ language proficiency. Future research may consider using longitudinal survey methods to track learners’ progress over time.

## Figures and Tables

**Table 1:** Coded Flanders Interactive Analysis System.

Classification		Code	Content
Teacher language	Indirect Impact	1	Acceptance of emotions
		2	Encouragement or praise
		3	Accepting or using students’ perspectives
		4	Asking a question
	Direct Impact	5	Lecture
		6	Giving guidance or instructions
		7	Criticizing or asserting authority
Student language		8	Students speaking passively
		9	Student-initiated speech
Silence or confusion		10	Invalid language

**Table 2:** Flanders classroom observation record form.

	Seconds	1	2	3	4	5	6	7	8	9	10	11	12	13	14	15	16	17	18	19	20
Minutes																					
1		10	10	6	4	9	4	9	4	3	4	9	3	9	9	2	4	8	3	3	6
2		8	4	8	6	8	6	6	8	3	6	8	6	8	8	6	8	4	9	4	9
3		3	6	5	6	5	8	6	5	5	2	9	5	5	8	5	5	5	9	4	9
4		5	9	8	5	5	6	8	9	4	4	8	5	8	4	9	5	9	5	2	6
5		8	5	8	9	5	8	6	8	5	9	8	6	5	8	6	8	5	8	4	9
6		5	8	6	8	5	5	9	5	6	9	9	2	5	5	8	6	5	4	9	5
7		5	6	10	6	6	9	9	9	9	4	6	6	10	8	8	8	3	4	8	10
8		10	4	8	8	8	4	8	2	6	10	8	8	8	2	4	8	8	8	3	4
9		4	9	9	4	5	5	6	2	6	8	8	8	2	4	8	4	5	8	8	4
10		5	8	5	8	9	4	8	8	5	8	8	8	6	5	8	5	8	5	2	4
11		9	6	9	2	9	2	9	2	9	10	2	4	9	5	8	5	8	6	8	6
12		4	4	8	6	8	5	8	5	8	8	6	10	10	5	5	8	5	8	6	10
13		9	3	8	5	8	5	9	9	3	6	8	5	6	8	8	8	8	8	8	8
14		2	8	8	8	8	8	8	8	5	5	8	8	6	5	8	5	8	5	8	5
15		8	5	8	5	8	2	9	4	9	3	6	8	5	5	4	9	5	8	6	8
16		4	9	4	9	9	8	6	9	4	5	8	8	4	9	5	9	5	8	9	8
17		8	9	5	8	6	9	8	5	8	9	8	9	6	8	5	9	8	6	5	8
18		5	8	5	6	10	4	9	6	10	10	4	9	9	9	9	9	6	8	3	5
19		8	8	3	8	3	8	8	6	10	4	8	8	8	5	8	8	5	8	2	4
20		9	4	8	9	3	3	4	9	8	3	6	8	8	6	9	9	9	9	9	9
21		9	9	4	8	8	6	6	6	10	10	9	9	9	8	8	3	4	6	10	10
22		9	9	9	9	9	9	9	9	9	9	5	8	2	6	6	6	6	6	9	9
23		9	9	9	9	9	9	9	9	6	6	8	8	6	8	8	8	2	6	4	9
24		4	9	4	9	4	4	9	6	6	4	10	10	5	5	5	5	5	5	5	5
25		5	5	5	5	5	5	5	5	5	5	5	5	5	5	5	5	5	5	5	5
26		5	5	10	4	4	9	9	4	8	4	9	9	6	10	5	5	5	5	4	9
27		9	6	8	8	10	5	5	5	5	4	9	6	8	6	8	8	6	5	5	5
28		5	4	8	5	8	5	8	4	3	9	4	8	8	2	5	5	5	5	4	8
29		8	5	6	8	8	10	5	5	5	4	9	9	8	8	4	9	4	9	4	9
30		6	5	5	4	9	4	9	8	4	9	3	6	8	5	8	6	6	6	6	5
31		10	10	10	10	10	10	2	6	10	10	10	4	6	6	8	8	8	8	8	8
32		8	2	6	5	5	8	4	4	4	9	4	9	2	5	5	4	9	2	2	5
33		4	9	9	2	2	5	4	9	6	8	8	5	5	5	5	4	8	8	8	8
34		8	8	2	6	8	8	8	8	8	8	8	8	8	8	8	8	6	6	6	10
35		10	10	10	10	10	10	10	10	4	4	6	10	2	9	3	10	2	6	6	10
36		8	8	8	8	8	8	8	8	8	8	8	8	8	8	8	8	8	8	8	8
37		8	2	2	4	9	6	10	10	10	10	10	10	10	10	10	10	10	10	10	10
38		10	10	10	10	10	10	10	10	10	10	10	10	10	10	10	4	9	2	4	9
39		9	4	9	6	6	6	6	9	9	9	6	6	6	6	4	6	10	6	6	10
40		10	6	6	10	10	10	10	10	10	10	10	10	10	10	10	10	10	10	10	10
41		10	10	10	10	10	10	10	10	10	10	10	10	10	10	10	10	10	10	10	10
42		10	10	10	10	10	10	10	10	10	10	6	6	4	6	6	10	4	8	2	3
43		8	8	8	8	8	8	8	8	8	8	8	8	8	8	8	8	8	8	8	8
44		8	8	8	8	8	8	8	8	2	2	2	4	9	4	9	9	4	9	9	2
45		2	5	9	9	9	9	9	4	9	9	2	5	5	9	3	6	6	10	6	6

**Table 3:** Matrix model table.

	1	2	3	4	5	6	7	8	9	10	Total
1	0	0	0	0	0	0	0	0	0	0	0
2	0	6	1	9	7	9	0	1	6	0	39
3	0	0	2	5	1	8	0	4	2	1	23
4	0	0	2	8	4	6	0	19	49	1	89
5	0	3	0	12	62	8	0	44	10	2	141
6	0	1	0	6	12	30	0	31	8	21	109
7	0	0	0	0	0	0	0	0	0	0	0
8	0	14	9	14	36	29	0	118	8	3	231
9	0	11	9	26	14	14	0	11	54	1	140
10	0	4	0	9	5	6	0	3	3	97	127
Total	0	39	23	89	141	110	0	231	140	126	899

**Table 4:** Classroom structure analysis.

Category	Number of Occurrences	Ratio	Time
Teacher Language	402	44.72%	20.1 min
Student Language	371	41.27%	18.6 min
Invalid Language	126	14.01%	6.3 min

**Table 5:** Analysis of the types of teacher control.

Class Type	Code	Projects	Number of Occurrences	Ratio		Indirect Language/ Direct Language
Indirect Impact	1	Acceptance of students’ emotions	0	0	37.57%	60.18%
	2	Expressing emotions to praise students	39	9.71%		
	3	Accepting or using the student’s claim	23	5.72%		
	4	Asking a question	89	22.14%		
Direct Impact	5	Lecture	141	35.07%	62.43%	
	6	Giving guidance or instructions	110	27.36%		
	7	Criticizing students or asserting authority	0	0		

**Table 6:** Analysis of teacher reinforcement types.

Category	Positive Reinforcement (Columns 1–3)	Negative Reinforcement (Columns 6–7)	Positive Reinforcement/ Negative Reinforcement
Number of times	62	110	56.36%

**Table 7:** Teacher–student closed-ended question and answer form.

Category	(4, 4)	(4, 8)	(8, 4)	(8, 8)	Total
Frequency	8	19	14	118	159
Ratio	0.89%	2.11%	1.56%	13.13%	17.69%

**Table 8:** Open-ended question and answer table for teachers and students.

Category	(3, 3)	(3, 9)	(9, 3)	(9, 9)	Total
Frequency	2	2	9	54	67
Ratio	0.22%	0.22%	1.00%	6.01%	7.45%
Category	(4, 4)	(4, 9)	(9, 4)	(9, 9)	Total
Frequency	8	49	26	54	137
Ratio	0.89%	5.45%	2.89%	6.01%	15.24%

## Data Availability

Data will be made available upon request.
